# Transforming Virtual Team-Based Learning for Rural Healthcare Staff: What the Pandemic Taught Us

**DOI:** 10.1093/geroni/igab046.2734

**Published:** 2021-12-17

**Authors:** Eve Gottesman, Helen Fernandez, Judith Howe

**Affiliations:** 1 US Department of Veterans Affairs, Bronx, New York, United States; 2 Icahn School of Medicine at Mount Sinai, New York, New York, United States; 3 James J. Peters VA Medical Center, Bronx, New York, United States

## Abstract

During COVID-19, many training programs pivoted to virtual formats. For the Rural Interdisciplinary Team Training (RITT) Program, funded by the Veterans Health Administration as part of the Geriatric Scholars Program, there were unique challenges. Given a history of successful accredited in-person, team-based workshops for staff at rural and remote clinics, program developers needed to quickly devise a plan for an effective virtual training for team members working separately from each other. Without the ability to provide in-person education and training, rapid pivoting to virtual modalities was essential for ongoing education of those providing care for older adults. Using a web-based platform, team members and expert trainer facilitation, participants engaged in lively discussions and reflection using the chat feature. RITT adapted the curriculum to better meet the needs of busy healthcare providers working during the pandemic, including increased discussion of how COVID affects older Veterans. Three virtual RITT workshops were held between March 2020 and February 2021 with 64 participants from 12 rural clinics and medical centers. Over 90% of participants agreed or strongly agreed that they were satisfied with the virtual workshop, comparable to those participating in the in-person workshop in earlier years. Similar to others, we have found that the ability to flex a curriculum has benefits to both learners and educators and increases the reach of educational opportunities in gerontology and geriatrics. Particularly in rural areas where travel may be challenging, a virtual format may be a desirable long-term solution for the RITT program.

